# Comparative Analysis of Putative Orthologues of Mitochondrial Import Motor Subunit: Pam18 and Pam16 in Plants

**DOI:** 10.1371/journal.pone.0078400

**Published:** 2013-10-23

**Authors:** Xuejin Chen, Bushra Ghazanfar, Abdul Rehman Khan, Sikandar Hayat, Zhihui Cheng

**Affiliations:** College of Horticulture, Northwest A&F University, Yangling, Shaanxi, China; Wilfrid Laurier University, Canada

## Abstract

Pam18/Tim14 and Pam16/Tim16, highly conserved proteins among eukaryotes, are two essential subunits of protein import motors localized in the inner mitochondrial membrane. The heterodimer formed by Pam18 and Pam16 via their J-type domains serves a regulatory function in protein translocation. Here, we report that thirty-one Pam18 and twenty-six Pam16 putative orthologues in twelve plant species were identified and analyzed through bioinformatics strategy. Results data revealed that Pam18 and Pam16 were also highly conserved among plants including their J-type domains within the hydrophilic region. Key amino acid residues and an HPD motif of Pam18 were identical among the orthologues except OsPam18L5. N-myristoylation sites of Pam18 and casein kinase II phosphorylation sites of Pam 16 were more abundant, which might be important functional sites. Some Pam18 and Pam16 proteins contained a transmembrane region at the N-terminal region. Sub-cellular prediction results indicated that many orthologues localized at mitochondria. Gene expression analyses revealed that Pam18 and Pam16 in *Arabidopsis* might play roles in senescence and abiotic stress responses. Our detailed study provides a better understanding of Pam18 and Pam16 in plant kingdom.

## Introduction

Mitochondria are essential organelles in eukaryotes, serving as a site of many biological processes, such as respiration, metabolism, development, calcium signaling, production of reactive oxygen species (ROS), cell death and so on [1-3]. Since more than 98 % of mitochondrial proteins are encoded by nuclear genes and synthesized on cytosolic ribosomes, the translocation across the outer and inner membranes is believed to play an important role in maintaining properly functioning mitochondria [4-7].

In mitochondrial translocation, there are two proteinaceous channels formed by the translocase of the outer membrane (TOM complex) and the presequence translocase of the inner membrane (TIM23 complex), respectively [8]. Mitochondrial preproteins destined for the matrix are directed by pre-sequences across both mitochondrial membranes [6]. In this process, pre-sequence translocase-associated import motor (PAM) complex, which localizes to the inner mitochondrial membrane, is critical for protein translocation into the matrix [9,10]. Mitochondrial HSP70 (mtHSP70), an ATP-dependent heat shock protein, is the core of the PAM complex [11-13]. Besides mtHSP70, there are four other subunits which have been analyzed in detail. Mge1 and the peripheral inner membrane protein Tim44, which serves as a binding site for mtHsp70, bringing it close to the protein import channel [8]. An inner membrane protein Pam18/Tim14 with a classical J domain stimulates the ATPase activity of mtHsp70 via its co-chaperone activity [8]. Pam16/Tim16 containing degenerate J domain (J-like domain) is dynamic and serves as a critical role in regulating the aforementioned ability of Pam18 [9,14,15]. Pam17, a nonessential component, was also found as part of the import motor [16,17].

Investigation has depicted that Pam18 and Pam16 can form a heterodimer via their J-type domains [18]. This interaction has been proposed to perform a critical regulatory functions in ATPase stimulatory activity of Pam18 [19]. One study revealed that Pam18 was reported to be not sufficient for its function within the import motor, but on the other hand, the regulation of its activity is equally important [20]. Based on a higher oligomeric state and structure of the heterodimer, the regulatory process is mediated by Pam16 [20]. On the whole, both Pam18 and Pam16 are essential components of the mitochondrial import motor and may serve a regulatory function in preprotein translocation.

All of the five critical components of this PAM complex are highly conserved among eukaryotes [21,22]. Homologues of yeast (*Saccharomyces cerevisiae*) ScPam18 and ScPam16 were identified in humans, DNAJC19 and Magmas respectively, which are associated with several human disorders [23]. In plants, many orthologues of proteins in the mitochondrial translocation system, including Pam18 and Pam16, have recently been investigated [24].

Although Pam18 and Pam16 are highly conserved in eukaryotes, their functions in plants are still unknown. Is it possible that Pam18 and Pam16 in plants play the similar role compared with yeast? This interesting notion acted as catalyst for this study, in which bioinformatics analysis for Pam18 and Pam16 orthologues from twelve representative plant species was performed. Thirty-one Pam18 and twenty-six Pam16 putative orthologues were identified and analyzed. Principle investigation was focused on their phylogenetic relationship, multiple alignment, amino acid composition, functional sites, hydrophobicity, transmembrane region, sub-cellular locations and expression patterns in *Arabidopsis thaliana* so as to better understand the roles of Pam18 and Pam16 in the plant kingdom with an objective to help elucidating their precise functions in the future.

## Materials and Methods

### Database Mining for Identification of Putative ScPam18 and ScPam16 Orthologues

ScPam18 and ScPam16 amino acid sequence were obtained from *Saccharomyces cerevisiae* database (http://www.yeastgenome.org/). To collect the orthologues of plants, amino acid sequences of ScPam18 and ScPam16 were used as query sequences to perform BLASTP searches [25] from National Center for Biotechnology Information (NCBI) non-redundant protein database (http://www.ncbi.nlm.nih.gov/). Organism was confined in plant species to narrow down the searching coverage. Sequences with *E* values above 1e-5 [26] and maximum identity less than 30 % were excluded from the dataset. Self-BLAST of the sequences was carried out manually to remove the redundancy. Each predicted Pam18 and Pam16 orthologues were reconfirmed using Pfam (http://pfam.sanger.ac.uk/search), SMART (http://smart.embl-heidelberg.de/) and CDD (http://www.ncbi.nlm.nih.gov/Structure/cdd/wrpsb.cgi).

### Multiple Sequence Alignment and Phylogenetic Tree Construction

Complete amino acid sequences were downloaded from NCBI database in FASTA format and alignments were performed using ClustalW. Subsequently, alignments were adjusted using Bioedit 7.0 software with 60 % threshold for homology. Full length protein sequence was employed to generate phylogenetic tree by the neighbor-joining method using MEGA 5.0 software [27]. The bootstrap test was carried out with 1000 replicates to assess the reliability of the interior nodes.

### Amino Acid Composition and Hydrophobicity Analyses

Amino acid composition and hydrophobicity analyses were carried out by Bioedit 7.0 software. Molar percentage of each residue in the sequence was copied to Excel to make histogram. Mean Hydrophobicity profiles were generated using the general method of Kyte and Doolittle with aligned sequences [28].

### Transmembrane Region Prediction and Functional Sites Analyses

TMpred program was used to predict transmembrane region. The algorithm is based on the statistical analysis of TMbase, a database of naturally occuring transmembrane proteins (http://www.ch.embnet.org/software/TMPRED_form.html). Online server SVMtm transmembrane domain predictor [29] (http://ccb.imb.uq.edu.au/svmtm/SVMtm_Predictor.shtml) was employed to ensure the quality of predication results. Amino acid sequences from thirty-one Pam18s and twenty-six Pam16s were submitted to search for functional sites using ScanProsite [30](http://prosite.expasy.org/scanprosite/).

### Prediction of Pam18 and Pam16 Sub-cellular Localization

Multiple predication servers were employed to predict the sub-cellular location of Pam18 and Pam16. TargetP 1.1 Server (http://www.cbs.dtu.dk/services/TargetP/) was based on N-terminal amino acid sequences for prediction [31]. Pam18 and Pam16 amino acid sequences in FASTA format were submitted to the website. Organism group was set to plant without specificity cutoffs. MitoProt II (http://ihg.gsf.de/ihg/mitoprot.html) calculates the N-terminal protein region to predict mitochondrial imported proteins. PSORT II (http://psort.hgc.jp/form2.html) based on both known sorting signal motifs and some correlative sequence features was used to make predictions.

### Microarray Expression Data Analyses

To identify genes coding AtPam18 and AtPam16, BLASTP using ScPam18 and ScPm16 as query sequences was carried out from TAIR (http://www.arabidopsis.org/) database. Protein sequences obtained from TAIR were performed BLASTP to reconfirm the genes. Putative genes coding AtTim44, AtTim23, AtTim17 and AtTim50 were identified through the same strategy. Microarray expression profiles were obtained from Genevestigator [32] (Data of Columbia-0 wild type were used only). Detailed experimental information can be acquired at the website (https://www.genevestigator.com/gv/).

### Plant Growth Conditions and Experiment Descriptions

Seeds of *Arabidopsis* plants (Columbia-0) were germinated and grown on 1/2 MS media in climate-controlled chambers under long day conditions (16 h light/8 h dark cycle) at 22 °C. Plants were transplanted to pots and leaf tissue was collected at different developmental stages. Seedlings, developed rosette, flowers and siliques, and senescence were designated to approximate 14, 21, 28 and 42 days old plants. Rosette leaves turned slightly yellow at the senescence stage. Abiotic stress treatments including heat (38 °C, 4 h), cold (4 °C, 4 h), salt (150 mM NaCl, 3 h) and drought (Dry air blow, 3 h) abiotic stress treatments were carried out when the plants were 28-day-old. Green tissues were harvested after treatments. All plant material was frozen in liquid nitrogen and stored at -80 °C before processing.

About 100 mg tissues were employed to extract total RNA using Column Plant RNAout reagent kit according to manufacturer’s protocol (TIANDZ, Beijing, China). RNA concentration was measured by NanoDrop2000 (Thermo SCIENTIFIC, USA) to further normalize RNA template among different samples. The first strand cDNAs were synthesized (~0.4 μg RNA as template) using SuperQuickRT cDNA Synthesis kit (CWBIO, Beijing, China). Real-time quantification RT-PCR reactions were performed in iQ^TM^5 machine (Bio-Rad, USA) using the SYBR *Premix Ex Taq* II (TaKaRa, Dalian, China) according to the manufacturer’s instructions. Each PCR reaction (20 μl) contained 10 μl Mix, 0.8 μl of each primer, and appropriately diluted cDNA. The PCR program was 95 °C for 30 s followed by 40 cycles of 95 °C for 20 s, 56 °C for 30 s, and 68 °C for 45 s. The *Actin-2* gene was used as internal reference for all the RT-PCR analysis. Each treatment was repeated three times independently.

## Results and Discussion

### Identification of ScPam18 and ScPam16 Orthologues in Plants

Previously, Pam18 and Pam16 of model organism *Saccharomyces cerevisiae* were identified as essential subunits of PAM complex (mitochondrial import motor). Amino acid sequences of ScPam18 and ScPam16 were used as query sequence to screen candidate orthologues in twelve representative plants: *Arabidopsis thaliana* (At), *Oryza sativa* (Os), Zea mays (Zm), Glycine max (Gm), Sorghum bicolor (Sb), *Solanum lycopersicum* (Sl), *Medicago truncatula* (Mt), *Populus trichocarpa* (Pt), *Vitis vinifera* (Vv), *Picea sitchensis* (Ps), *Brachypodium distachyon* (Bd) and Physcomitrella patens (Pp). In all, thirty-one Pam18 and twenty-six Pam16 (Table S1) non-redundant proteins were identified and their uniqueness was manually verified by removing redundant sequences from our dataset. They are named as Pam18 and Pam16 in brief for convenience except for some special claims. These proteins were named according to their genus followed by “L” meaning like, and numbered 1 through 5 to represent *E* values in ascending order from low to high since there was no standard nomenclature assigned to these newly-identified orthologues. Protein length of Pam18s ranges from 76 to 166 amino acids and Pam16s ranged from 108 to 345 amino acids, indicating Pam16 is larger than Pam18 in general. Compared with other species, more orthologues were identified from *Oryza sativa*, *Solanum lycopersicum* and *Vitis vinifera* (Table S1).

The selected plant species include lower plant, monocot, eudicot and xylophyta, which can serve as representatives for other closely related species. Pam16 orthologue (not shown) was also identified in *Lotus japonicas* and *Solanum tuberosum* respectively. However, no Pam18 orthologues could be identified from the two species based on our BLASTP search. This circumstance may suggest that Pam16 is more universal when compared with Pam18 in the plant kingdom. Orthologues of Pam18 and Pam16 were also identified in *Arabidopsis thaliana* and *Solanum lycopersicum* in previous study [24]. As an increasing number of plant species are being sequenced [33,34], more orthologues may be identified in the future. As mentioned above, since Pam17 is also a subunit of PAM, data mining was performed to screen for orthologues. However, we could not identify candidate orthologues as reported previously [24].

### Phylogenetic Relationship of Pam18 and Pam16 in Plants

**Figure 1:**
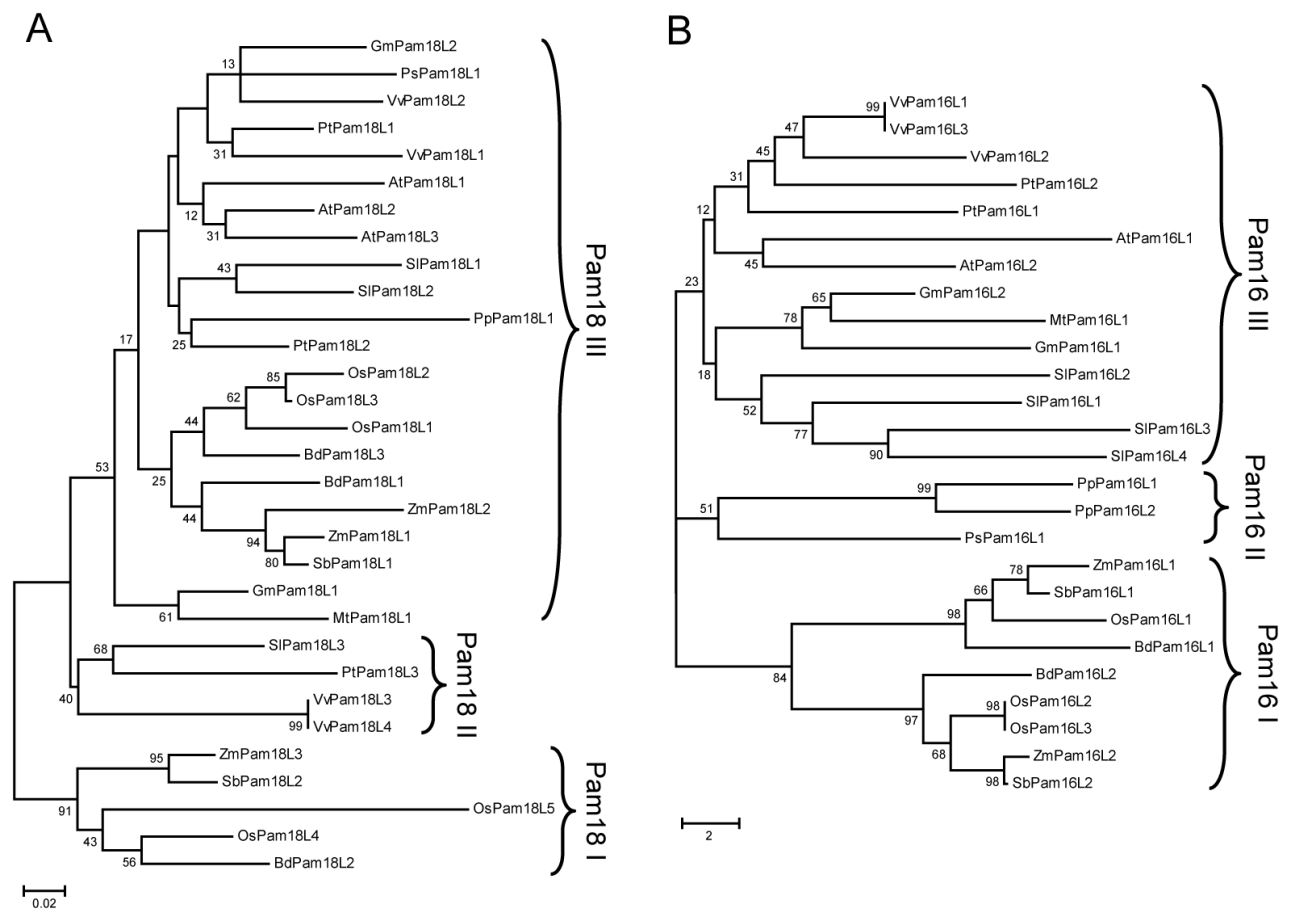
Phylogenetic tree analysis of putative Pam18 (A) and Pam16 (B) orthologues from twelve plant species. Both Pam18 (A) and Pam16 (B) phylogenetic trees were generated by the neighbor-joining method and bootstrap values (1000 replications) are indicated at each branch. The unit bar on the tree represents the measure of phylogenetic distance.

Pam18 and Pam16 as conserved proteins have several orthologues in plants, however, their phylogenetic relationship is not clear. To investigate their evolutionary history in plants, phylogenetic analysis was carried out. Phylogenetic analysis indicated that both Pam18 and Pam16 can be divided into three major sub-groups (Figure 1). Proteins from *Gramineae* plants: OsPam18L4, OsPam18L5, ZmPam18L3, SbPam18L2 and BdPam18L2 belonged to Pam18I sub-group. Pam18II included SlPam18L3, PtPam18L3, VvPam18L3 and VvPam18L4. Other Pam18 orthologues including eight gramineous members were grouped into Pam18III (Figure 1A). All gramineous members of Pam16 belonged to Pam16I sub-group. PpPam16L1, PpPam16L2 and PsPam16L1 were grouped into Pam16II, and Pam16III contained other orthologues (Figure 1B).

The phylogenetic tree indicated that some proteins display extremely high similarity and form pairs with each other including two pairs from different species, *Zea mays* and *Sorghum bicolor* (VvPam18L3 and VvPam18L4; ZmPam18L3 and SbPam18L2; VvPam16L1 and VvPam16L3; PpPam16L1 and PpPam16L2; OsPam16L2 and OsPam16L3; ZmPam16L2 and SbPam16L2). The phylogenetic relationship of Pam16 correlates with their evolutionary relationship among different plant species.

### Multiple Sequence Alignments of Pam18 and Pam16

**Figure 2:**
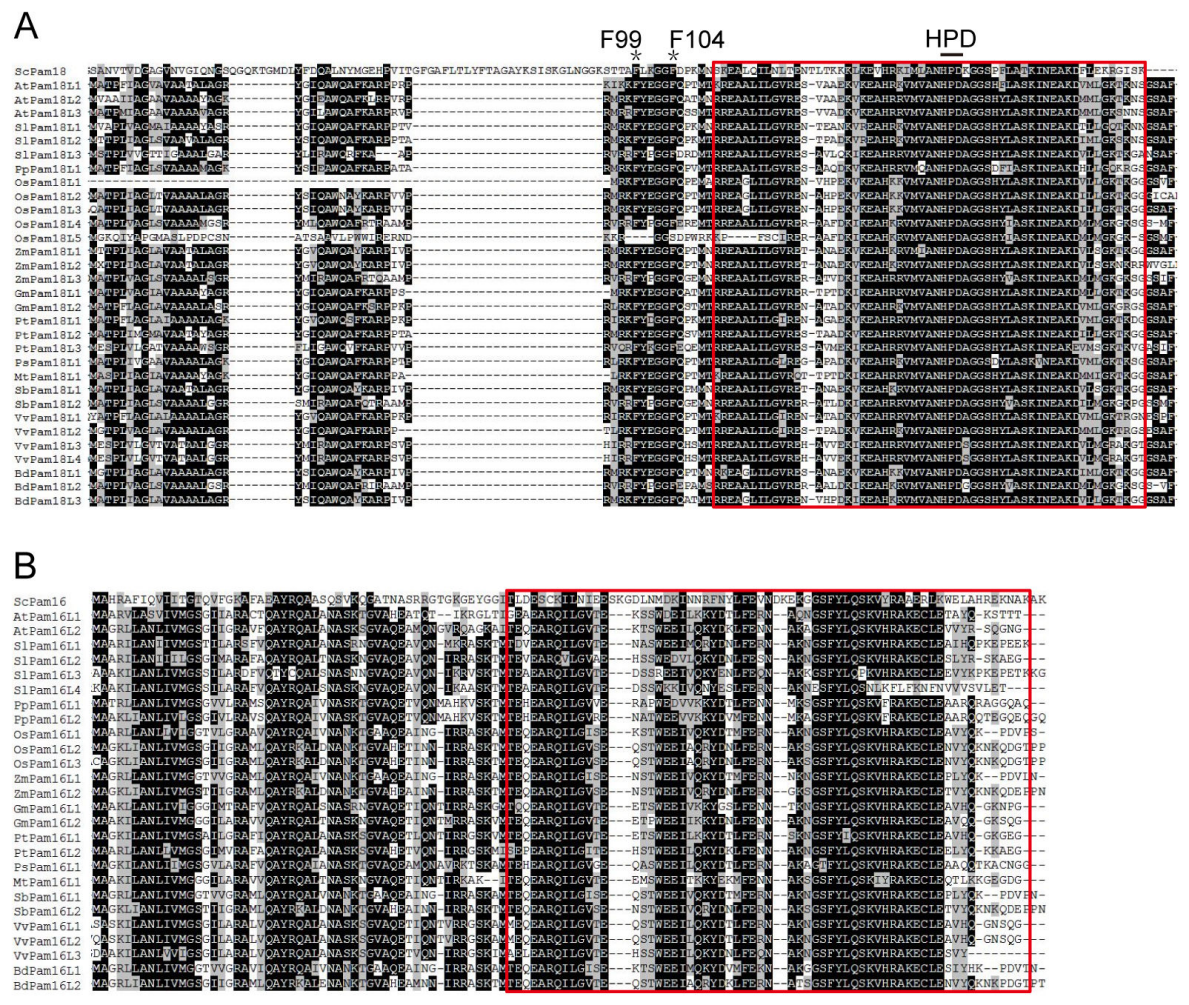
Amino acid sequence alignment of Pam18 (A) and Pam16 (B) in *Saccharomyces cerevisiae* and twelve plant species. Only the conserved regions are shown. Identical amino acid residues were shaded black and similar amino acid residues were shaded grey. J-domain of Pam18 (A) and J-like domain of Pam16 (B) are indicated in the red rectangle. An HPD motif is shown and two important phenylalanine residues are indicated by asterisk (A). ScPam18 and ScPam16 protein sequence were used for comparison.

Multiple sequence alignments were performed to analyze the conserved amino acid residues and domains of Pam18 and Pam16 among plants. The distributions of conserved amino acids of Pam18 and Pam16 are extremely similar among the members in twelve plant species with the exception of OsPam18L1 and OsPam18L5 (Figure 2). ScPam18 interacts with ScPam16 through their J-type domain, which was widely demonstrated to be critical in the regulation of protein translocation [35,36]. According to Pfam, SMART and CDD databases, all the orthologues of Pam18 contain a J-domain (Figure 2A) and orthologues of Pam16 contain a J-like domain (Figure 2B). The J-type domains of Pam18 and Pam16 in plants are all located in the C-terminal region. ScPam18, a 168 amino-acid protein has a critical motif HPD in the C-terminal matrix-localized J-domain (Figure 2A, Figure S1A). This invariant HPD motif was involved in stimulating mtHsp70 activity [37,38]. Compared with ScPam18, all the orthologues in plants include an HPD motif (Figure 2A). The region (aa 99-109) of ScPam18 has been identified to be an important arm for its function [19] (Figure S1A), including two important residues, F99 and F104 respectively (Figure 2A). There is a similar region among orthologues of plants and two phenylalanine residues are identical with the exception of OsPam18L5 (Figure 2A).

Taken together, except OsPam18L1 and OsPam18L5, Pam18 and Pam16 orthologues among plants show highly conserved amino acid sequences and contain key HPD motif and residues compared with ScPam18 and ScPam16.

### Analysis of Amino Acid Composition and Functional Sites

**Figure 3:**
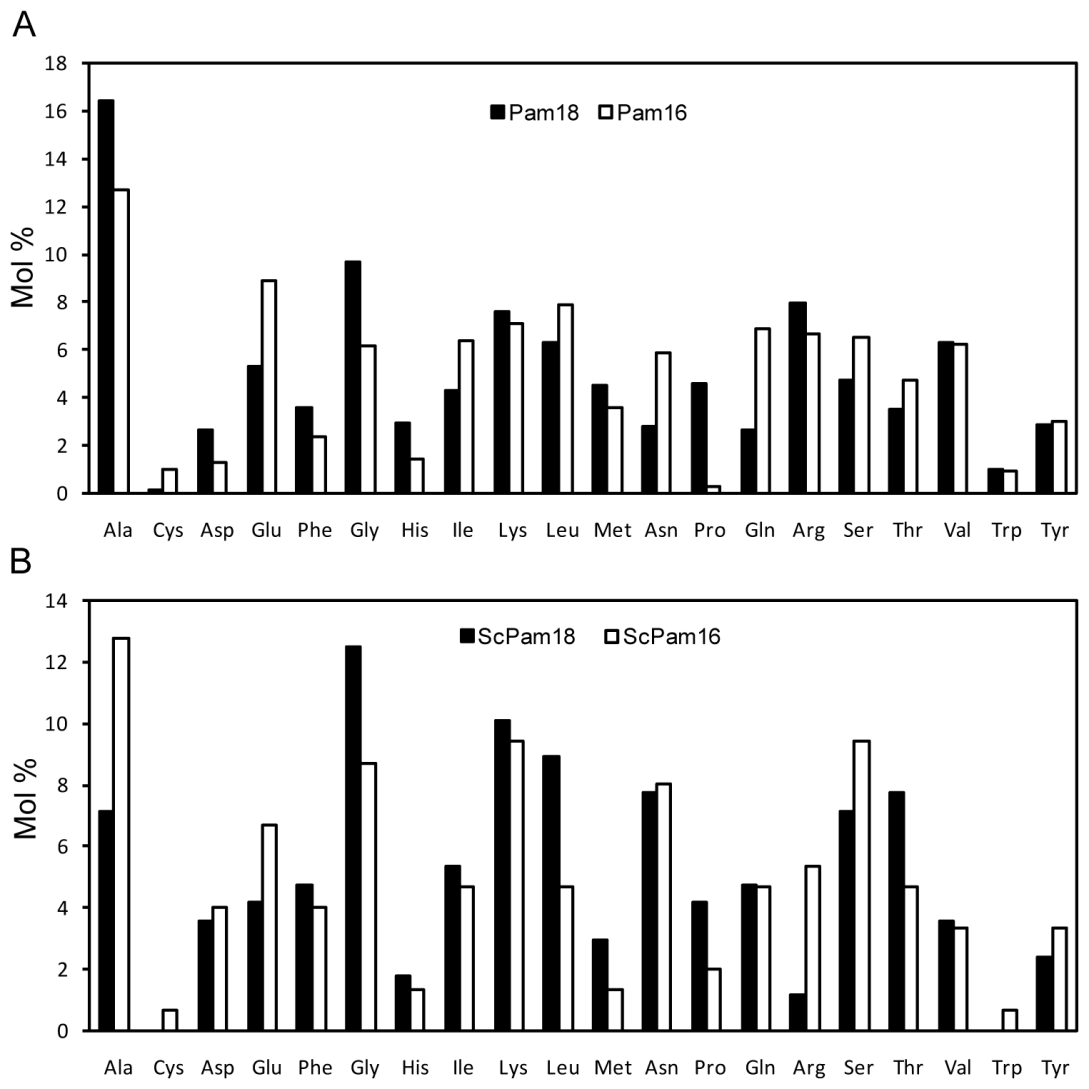
Amino acid composition of Pam18 and Pam16 in twelve plant species (A) and *Saccharomyces cerevisiae* (B). Thirty-one amino acid sequences of Pam18 were combined together to evaluate average amino acid composition using Bioedit 7.0 software. The same strategy was adopted in Pam16 amino acid evaluation (Twenty-six sequences in total). Histograms show the Molar percent of each residue of the combined sequence. Amino acid composition of ScPam18 and ScPam16 is shown for comparison.

For proteins, biochemical properties of the various amino acids play an important role in their functions. Here, we used Bioedit 7.0 software to analyze amino acid composition. The content of two aliphatic-type amino acids, alanine and glycine, are higher in Pam18 and Pam16 (Figure 3A), which is consistent with ScPam18 and ScPam16 (Figure 3B). Alanine and glutamic acid are more abundant in Pam16 compared with other amino acids (Figure 3A). The content of cysteine and tryptophan are relatively lower in Pam18 and Pam16. There is no cysteine and tryptophan in ScPam18. Overall, alanine and glycine are more abundant in all investigated proteins.

To predict functional sites of Pam 18 and Pam 16, ScanProsite was used [30]. N-glycosylation, N-myristoylation and four types of phosphorylation sites were predicted in Pam18 and Pam16. According to prediction results, no tyrosine kinase phosphorylation site or cAMP- and cGMP-dependent protein kinase phosphorylation sites were observed. N-myristoylation sites were more abundant than other functional sites in Pam18. In Pam16, casein kinase II phosphorylation site was relatively more abundant. Tyrosine kinase phosphorylation site and cAMP- and cGMP-dependent protein kinase phosphorylation sites were less abundant.

In plants, the N-glycosylation of proteins has a great impact both on their physicochemical properties and on their biological functions [39]. N-terminal myristoylation plays a vital role in membrane targeting and signal transduction in plant responses to environmental stress [40]. Phosphorylation turns many protein enzymes on and off, thereby altering their function and activity. There is a very close relationship between phosphorylation and signaling, as well as metabolism in plants [41]. These functional sites especially abundant ones (N-myristoylation site of Pam18 for example), may be referred to important roles in Pam18 and Pam16 function.

### Hydrophobicity and Transmembrane Region Analysis

**Figure 4:**
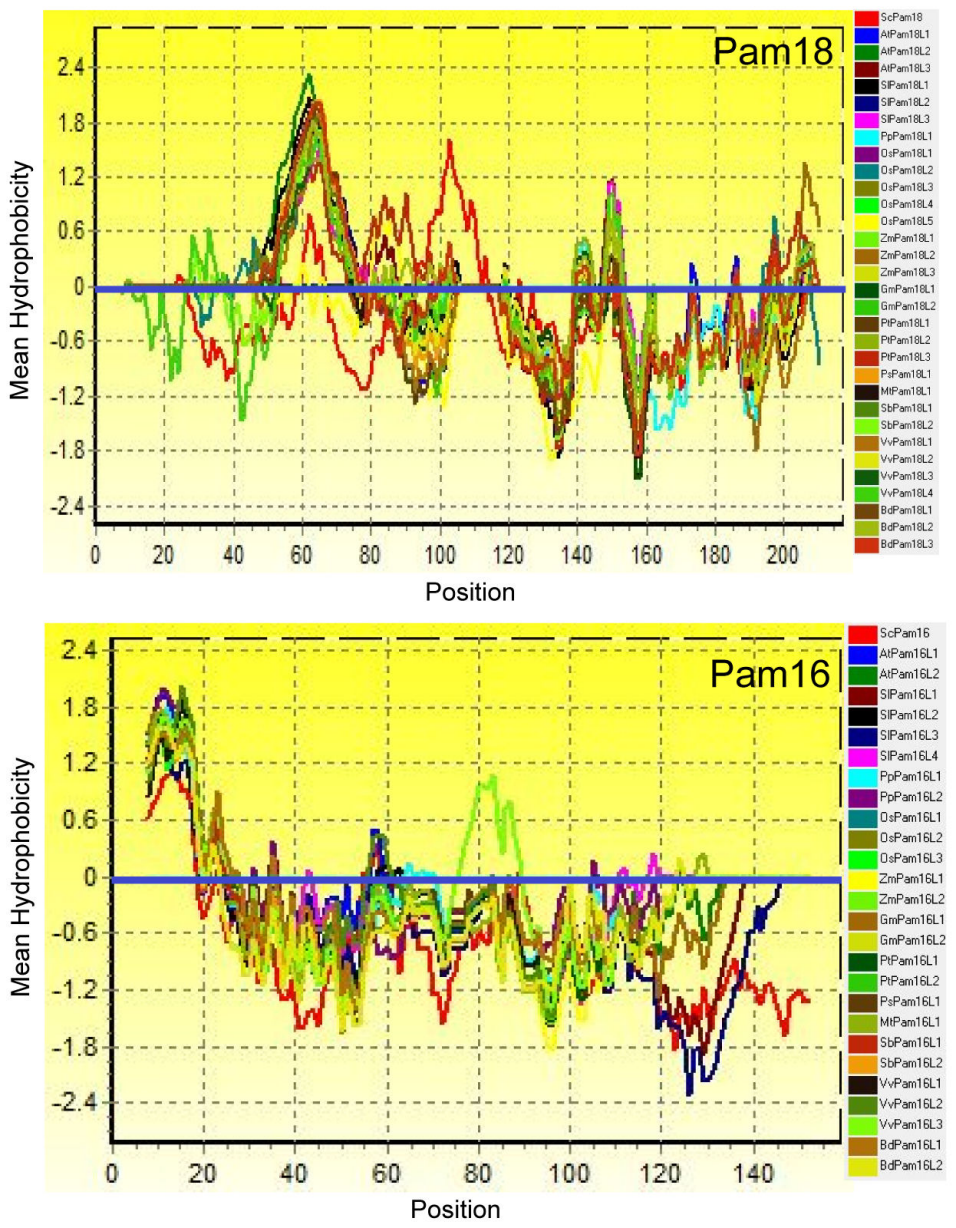
Mean Hydrophobicity profiles of Pam18 and Pam16. Thirty-one aligned amino acid sequences (Pam18) including ScPam18 and twenty-six aligned amino acid sequences (Pam16) including ScPam16 were used to evaluate hydrophobicity using Bioedit 7.0 software. ScPam18 and ScPam16 are indicated by a red line.

Hydrophobicity of a protein is determined by its amino acid sequence, which can be categorized as hydrophobic, polar, non-charged, non-aliphatic, acidic, or positively charged residues. In order to investigate the overall protein hydrophobicity, mean hydrophobicity profiles were generated. One obvious hydrophobic region (around 50-80 aa) in Pam18 was observed (Figure 4). Other areas of Pam18 including J-domain could be regarded as hydrophilic region (Figure 4). The region around 1-20 aa of Pam16 was hydrophobic and their J-like domains were within the hydrophilic region (Figure 4). Besides a C-terminal J-like domain, ScPam16 has a hydrophobic region at 1-28 aa (Figure S1A) consistent with Pam16. On the whole, the J-domain and J-like domain all correspond to hydrophilic regions, which is consistent with ScPam18 and ScPam16. The transmembrane (TM) region of these orthologues was also predicted using the TMpred program (Table 1). Except OsPam18L1 and OsPam18L5, all the other Pam18s and Pam16s were predicted to have transmembrane region (Table 1). Some of them contain two transmembrane regions, such as SlPam16L4, OsPam16L3 and VvPam16L2. To ensure the quality of these prediction results, SVMtm transmembrane domain predictor was adopted to reconfirm the prediction (Table 1). However, some orthologues were not predicted to have TM region according to this online predictor (Table 1). This difference could possibly be resulted from differed criterion between the two programs. TM regions of orthologues based on two prediction results were more reliable. The positions of their TM region were predicted as well.

**Table 1:** Transmembrane region prediction of Pam18 and Pam16.

**Orthologues**	**Number of transmembrane region/Position (aa)**	
	**TMpred prediction**	**SVMtm predictor**
AtPam18L1	1/1-18	0
AtPam18L2	1/1-16	0
AtPam18L3	1/2-18	1/4-18
SlPam18L1	1/1-17	1/2-16
SlPam18L2	1/1-18	0
SlPam18L3	1/1-22	0
PpPam18L1	1/1-17	0
OsPam18L1	0	0
OsPam18L2	1/21-38	1/24-38
OsPam18L3	1/1-20	0
OsPam18L4	1/1-18	0
OsPam18L5	0	0
ZmPam18L1	1/1-18	0
ZmPam18L2	1/1-18	0
ZmPam18L3	1/22-38	1/24-38
GmPam18L1	1/1-18	1/2-16
GmPam18L2	1/1-17	1/2-16
PtPam18L1	1/1-17	1/2-16
PtPam18L2	1/1-18	0
PtPam18L3	1/6-25	0
PsPam18L1	1/1-17	1/2-16
MtPam18L1	1/1-16	1/2-16
SbPam18L1	1/1-18	0
SbPam18L2	1/22-38	1/24-38
VvPam18L1	1/4-21	0
VvPam18L2	1/1-18	0
VvPam18L3	1/3-20	0
VvPam18L4	1/57-76	1/58-72
BdPam18L1	1/1-18	0
BdPam18L2	1/1-18	0
BdPam18L3	1/1-17	0
AtPam16L1	1/2-18	1/3-17
AtPam16L2	1/1-18	0
SlPam16L1	1/2-18	0
SlPam16L2	1/1-18	1/4-18
SlPam16L3	1/16-33	1/14-31
SlPam16L4	2/114-136, 168-188	2/115-129, 171-185
PpPam16L1	1/5-25	0
PpPam16L2	1/4-18	1/4-18
OsPam16L1	1/1-25	0
OsPam16L2	1/3-25	1/3-17
OsPam16L3	2/42-62, 230-254	2/41-55, 233-247
ZmPam16L1	1/1-22	0
ZmPam16L2	1/5-24	1/3-17
GmPam16L1	1/1-25	1/4-18
GmPam16L2	1/1-18	1/4-18
PtPam16L1	1/5-24	1/4-18
PtPam16L2	1/4-22	0
PsPam16L1	1/5-22	1/4-18
MtPam16L1	1/4-18	1/4-18
SbPam16L1	1/1-22	0
SbPam16L2	1/5-18	1/3-17
VvPam16L1	1/6-24	0
VvPam16L2	2/21-37, 142-159	1/127-155
VvPam16L3	1/4-21	0
BdPam16L1	1/1-24	0
BdPam16L2	1/1-24	1/3-17

Besides a C-terminal J-domain, ScPam18 has a single membrane-spanning region (Figure S1B). But the position of transmembrane region in ScPam18 (65-84 aa) is different from Pam18 (Table 1).There is no TM region in ScPam16 (Figure S1B). However, some Pam16 orthologues include one or two transmembrane regions (Table 1).

### Prediction of Sub-cellular Locations of Pam18 and Pam16

ScPam18 and ScPam16, subunits of PAM, localize to the mitochondrial inner membrane. Therefore, investigation was carried out for the sub-cellular locations of Pam18 and Pam16 in plants. TargetP was used to predict the sub-cellular location of eukaryotic proteins based on the predicted presence of any of the N-terminal presequences [31]. Some Pams can be considered to localize at mitochondria according to TargetP prediction results (Table 2). It is also possible for some Pams targeting at chloroplast, secretory pathway and other location (Table 2). The proportion of mitochondrial location was higher than other locations based on the orthologues investigated. The sub-cellular location of ScPam18 and ScPam16 was also predicted via TargetP. However, the prediction sites were not mitochondria, but other locations (not show). The possible reason may result from inaccurate prediction. To validate the reliability of prediction results, multiple online servers were employed to make predictions. Since mitochondrion is a probable destination of some Pams, MitoProt program was utilized because it calculates the N-terminal protein region that support a mitochondrial targeting sequence exclusively. The prediction results are shown in form of probability of export to mitochondria, relatively higher values indicate higher probability (Table 2). Similar results were acquired from another predictor, PSORT II (Table 2). Taken together, it is possible that some Pams localize at mitochondria according to three prediction systems. In addition, there is also a possibility that some Pams may function in other organelles in plants.

**Table 2:** Prediction of sub-cellular location of Pam18 and Pam16.

**Orthologues**	**TargetP prediction** ^a^	**MitoProt II prediction^b^**	**PSORT II prediction^c^**
AtPam18L1	S (5)	0.8488	13.0 % (2)
AtPam18L2	S (4)	0.3887	17.4 % (2)
AtPam18L3	C (5)	0.5581	43.5 % (1)
SlPam18L1	S (5)	0.6808	17.4 % (2)
SlPam18L2	S (5)	0.3997	21.7 % (2)
SlPam18L3	M (4)	0.9765	30.4 % (1)
PpPam18L1	S (4)	0.1966	17.4 % (3)
OsPam18L1	-(4)	0.4357	4.3 % (4)
OsPam18L2	-(5)	0.0548	26.1 % (2)
OsPam18L3	M (5)	N/A	N/A
OsPam18L4	M (4)	0.7798	22.2 % (2)
OsPam18L5	-(5)	0.0281	4.3 % (3)
ZmPam18L1	M (4)	0.7982	N/A
ZmPam18L2	M (4)	0.7914	N/A
ZmPam18L3	-(3)	0.0638	17.4 % (2)
GmPam18L1	M (5)	0.5650	N/A
GmPam18L2	M (4)	0.7987	11.1 % (3)
PtPam18L1	S (5)	0.2903	N/A
PtPam18L2	M (5)	0.7479	N/A
PtPam18L3	M (5)	0.7902	17.4 % (2)
PsPam18L1	S (4)	0.2760	N/A
MtPam18L1	S (4)	0.5740	4.3 % (4)
SbPam18L1	M (4)	0.8030	N/A
SbPam18L2	-(3)	0.0577	21.7 % (2)
VvPam18L1	S (4)	0.8078	N/A
VvPam18L2	M (4)	0.3548	N/A
VvPam18L3	M (4)	0.7710	17.4 % (2)
VvPam18L4	-(3)	0.3267	17.4 % (2)
BdPam18L1	M (4)	0.7459	N/A
BdPam18L2	M (4)	0.8170	11.1 % (3)
BdPam18L3	M (5)	0.7357	N/A
AtPam16L1	M (3)	0.7934	30.4 % (1)
AtPam16L2	M (2)	0.7701	34.8 % (1)
SlPam16L1	M (3)	0.9387	65.2 % (1)
SlPam16L2	M (5)	0.9532	52.2 % (1)
SlPam16L3	S (4)	N/A	N/A
SlPam16L4	-(1)	0.0034	26.1 % (2)
PpPam16L1	M (4)	0.7333	43.5 % (1)
PpPam16L2	S (4)	0.5751	11.1 % (3)
OsPam16L1	S (4)	0.7703	34.8 % (1)
OsPam16L2	-(5)	0.7167	21.7 % (3)
OsPam16L3	M (4)	0.5750	30.4 % (2)
ZmPam16L1	M (5)	0.7990	30.4 % (1)
ZmPam16L2	-(5)	0.7659	30.4 % (2)
GmPam16L1	M (4)	0.8647	47.8 % (1)
GmPam16L2	S (5)	0.8101	17.4 % (2)
PtPam16L1	M (5)	0.8766	21.7 % (2)
PtPam16L2	M (4)	0.9461	34.8 % (1)
PsPam16L1	S (4)	0.8303	13.0 % (3)
MtPam16L1	S (5)	0.7890	17.4 % (2)
SbPam16L1	M (4)	0.7933	30.4 % (1)
SbPam16L2	-(5)	0.7659	30.4 % (2)
VvPam16L1	S (2)	0.8888	30.2 % (1)
VvPam16L2	S (5)	0.0813	13.0 % (3)
VvPam16L3	S (5)	0.6616	17.4 % (3)
BdPam16L1	S (5)	0.8022	30.4 % (1)
BdPam16L2	M (5)	0.7960	34.8 % (2)

^a^ S, Secretory pathway; C, Chloroplast; M, Mitochondrion; – Any other location.

Reliability class is in bracket, from 1 to 5, the lower the value, the more reliable the prediction.

^b^ Probability of export to mitochondria, relatively higher values indicate higher probability.

^c^ Percentage chance of mitochondrial location, values from 1 to 4 display percentage class compared with any other location.

### Expresssion Pattern of AtPam18 and AtPam16 in *Arabidopsis thaliana*

There are three orthologues of Pam18 (*AtPam18L1*/*At2G35795*, *AtPam18L2*/*At3G09700*, *AtPam18L3*/*At5G03030*) and two orthologues of Pam16 (*AtPam16L1*/*At5G61880*, *AtPam16L2*/ *AT3G59280*) in the model plant *Arabidopsis*. Besides Pam18 and Pam16, mitochondrial translocation complex also includes other import subunits, such as Tim44, Tim23, Tim17 and Tim50 in *Saccharomyces cerevisiae*. Putative genes coding these import components were identified as *AtTim44* (*At2G20510*, *At2G36070*), *AtTim23* (*At1G17530*, *At1G72750*, *At3G04800*), *AtTim17* (*At2G37410*) and *AtTim50* (*At1G55900*) in *Arabidopsis*.

**Figure 5:**
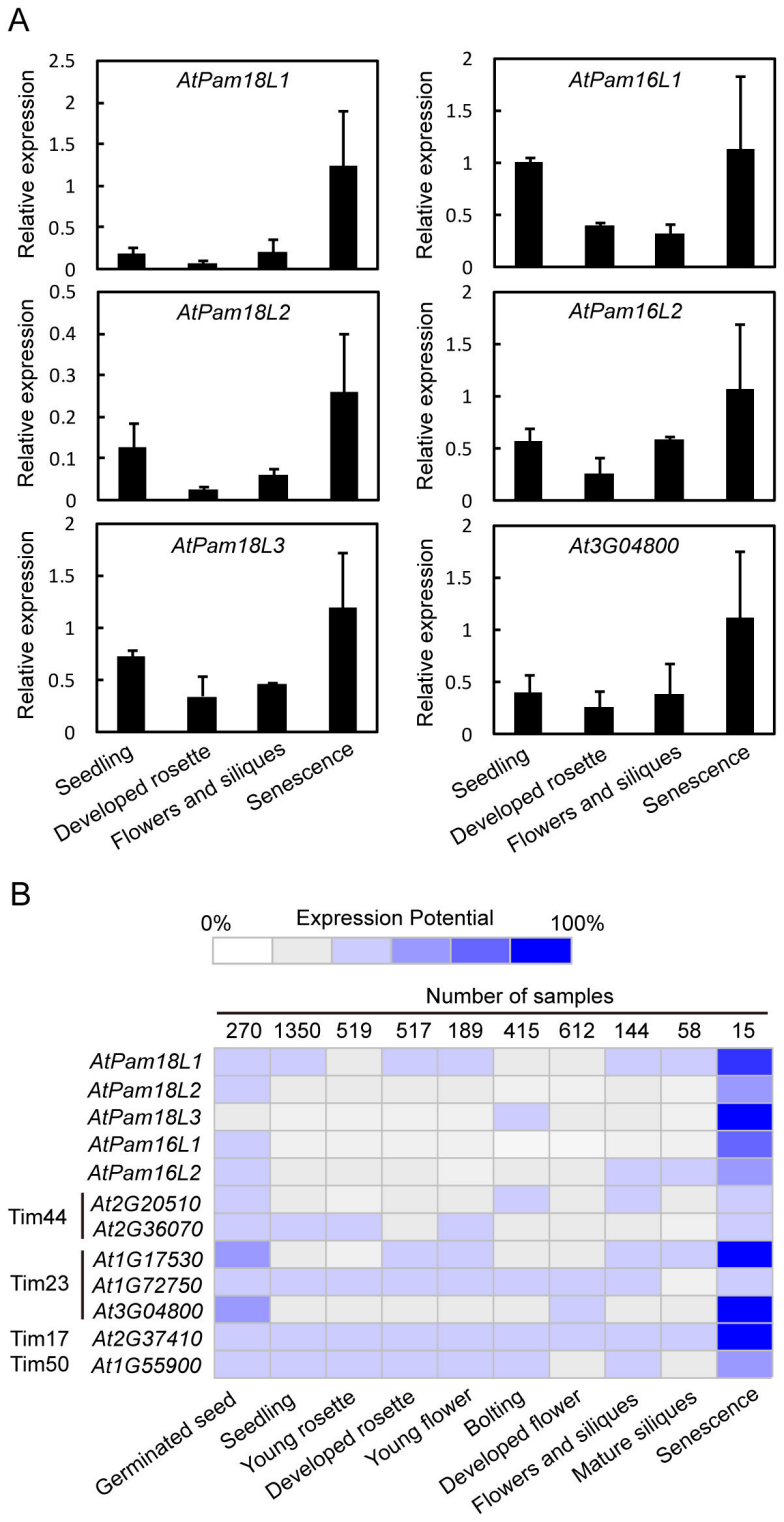
Expression pattern of *AtPam18* and *AtPam16* at developmental stages. Relative expressions of *AtPam* and *At3G04800* were determined by real-time qRT-PCR. Expression levels were normalized by *Actin-2*. Error bars represent means of three replicates ± SD. Similar results were obtained from three independent replicates and one representative result is shown (A). Expression profiles as heat map (B) in *AtPam* and putative genes coding AtTim44, AtTim23, AtTim17 and AtTim50 were generated by Genevestigator.

To analyze the expression patterns of these *AtPam* genes at different developmental stages, such as seedling, developed rosette, flowers and siliques, and senescence stage stages, real-time qRT-PCR was carried out. One of *AtTim23* (*At3G04800*) was chosen as a reference to determine if other subunit genes expressed similarly. As show in Figure 5A, *AtPam* genes and *At3G04800* were expressed during all developmental stages analyzed, but their expression levels differed. These genes were expressed relatively higher at senescence stage compared with previous developmental stages. At seedling stage, *AtPam18L2*, *AtPam18L3* and *AtPam16L1* showed relatively higher expression level compared with developed rosette and flower stages. On the whole, all genes investigated were expressed lower at developed rosette and flower stages (Figure 5A). To confirm the gene expression results, microarray data were obtained and analyzed from Genevestigator using standard heat map. Other import component genes were also investigated as reference. The expression potential of *AtPam* genes was much higher at the senescence stage, as well as *At1G17530*, *At3G48000*, *At2G37410* and *At1G55900* (Figure 5B). At other development stages, the expression potential of these genes was at the similar lower level (Figure 5B). The expression pattern results from real-time PCR and microarray are consistent to some extent. Higher level expression at senescence stage suggested *AtPam* genes to possibly play a role in plant aging.

**Figure 6:**
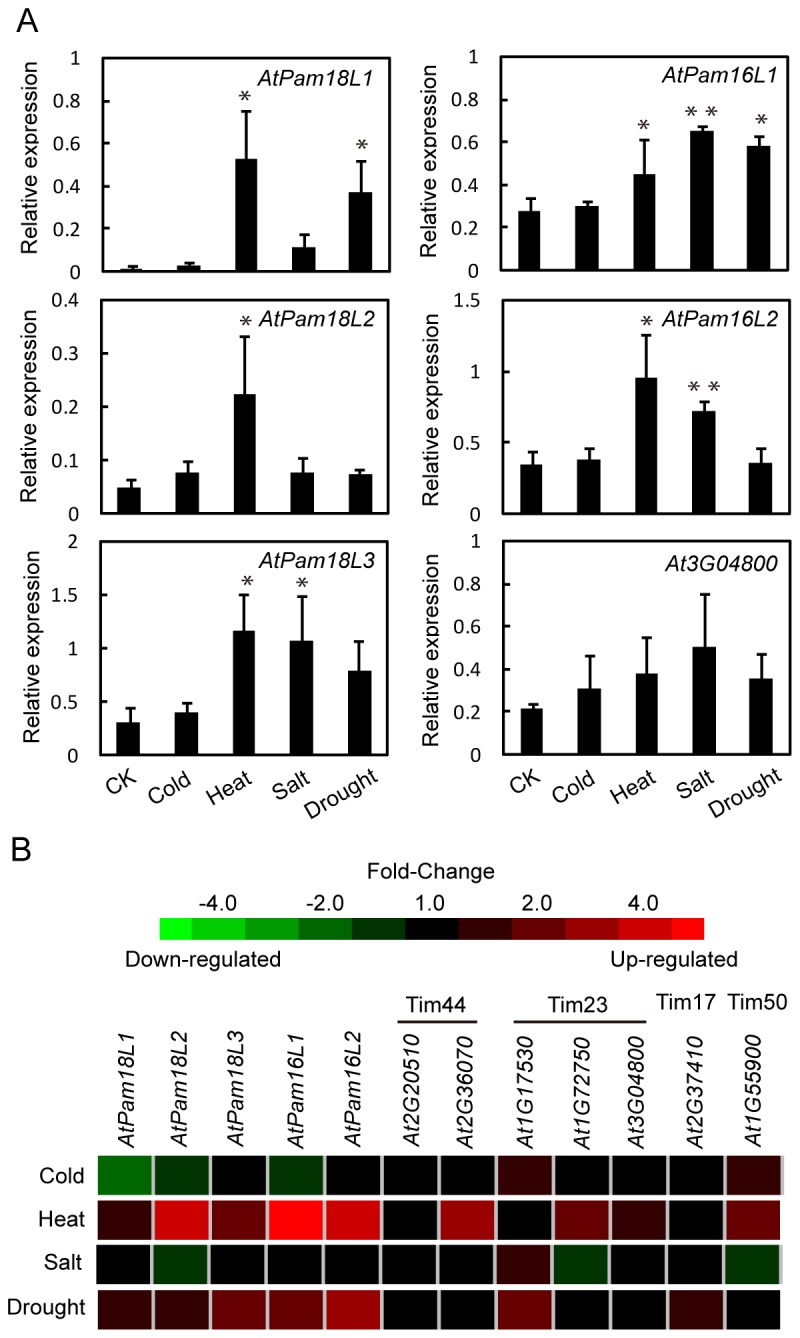
Expression pattern of *AtPam18* and *AtPam16* under cold, heat, salt and drought stresses. Relative expressions of AtPam and At3G04800 were determined by real-time qRT-PCR. Expression levels were normalized by Actin-2. Error bars represent means of three replicates ± SD. Asterisks indicate significant differences of cold, heat, salt and drought treatments compared with control based on Student’s t test, P<0.05 (*), P<0.01 (**). One of three independent experimental replicates with similar results is shown (A). Fold-change expression levels (B) in AtPam and putative genes coding AtTim44, AtTim23, AtTim17 and AtTim50 were from Genevestigator.From these results, it is speculated that AtPam genes might play key roles in plant senescence and heat stress responses.

Gene expression levels were analyzed after abiotic stress treatment to better understand gene function. As show in Figure 6A, there is no significant change in the gene expression after cold treatment compared with control. *AtPam18L1*, *AtPam18L2*, *AtPam18L3*, *AtPam16L1* and *AtPam16L2* were highly up-regulated after 4 hours heat treatment. Subjecting to salt stress, the expression level of *AtPam18L3* , *AtPam16L1* and *AtPam16L2* greatly increased. Drought stress can induce the expression level of *AtPam18L1* and *AtPam16L1*. Apart from *AtPam* genes, no expression variation was observed in *At3G04800*. Fold-change map obtained from Genevestigator show similar expression changes in heat stress treatment, but no significant variations were observed in cold, salt and drought treatment (Figure 6B). The conflicting results between real-time qRT-PCR and microarray database could possibly be due to the different growth conditions or different experimental conditions.

Plants, like other organisms, have both unintended and programmed aging. Senescence and programmed cell death (PCD) are important features for plant development. Through allowing nutrient recycling and reallocation in plant life, senescence contributes to the plant survival and the developmental program [42]. Some believe that senescence is one type of PCD that occurs in plants. A large number of senescence-associated genes (SAGs) have been identified in various plant species [43]. Studies on different SAGs reveal a diverse range of gene-activation patterns during senescence, indicating that plant senescence involves multiple regulatory pathways [44]. Mitochondria, a key organelle, is thought to be the energy source and involved in many biological processes, such as metabolism, calcium signaling, development and PCD etc. In this study, putative orthologues of mitochondrial import motor subunit Pam18 and Pam16 were identified in plants. According to gene expression analysis in *Arabidopsis*, *AtPam18* and *AtPam16*, as well as *AtTim23* and *AtTim17* were highly expressed at the senescence stage. It is therefore presumed that mitochondrial import translocation system may participate in plant aging.

Environmental stresses such as cold, heat, drought, and salinity greatly influence plant growth, development and productivity. Plants respond and adapt to these stresses at physiological, biochemical and molecular levels. Abiotic stress has been shown to induce the expression of genes with various functions in a variety of plants [45]. Mitochondria are a source of ROS which accumulate in plant cells when confront diverse environmental stress conditions. Plant mitochondria are proposed to act as signaling organelles in responses to biotic and abiotic stress [46,47]. Genes encoding mitochondrial proteins in response to stress have been identified in *Arabidopsis* [48]. In this study, gene expression analysis revealed that *AtPam18* and *AtPam16* were up-regulated after heat stress treatment. The expression of some *Pam* genes was induced by drought and salt stress. Genes coding other mitochondrial import components were not expressed in accordance with *AtPam* genes. The exact mechanism about how these subunits cooperate with each other remains largely unknown in mitochondrial import translocation. It is possible that mitochondrial import translocation system may be involved in abiotic stress responses.

In the perspective of protein domains, all AtPam18s are regarded as chaperone DnaJ-domain superfamily proteins, which function in heat shock protein binding and folding. The heat-induced expression level of *AtPam18s* may possibly be associated with their binding ability with heat shock proteins. All AtPam16s contain one degraded J domain, so it is possible that this domain has a similar function with DnaJ-domain. The high expression level of *AtPam16s* to heat stress may also result from their binding ability. The gene *AtPam16L2*/*AT3G59280* was first named as *TXR1* and was proposed as a regulator of a transport mechanism [49]. The mutant *txr1* exhibited increased resistance to thaxtomin, a phytotoxin secreted by bacteria in the genus *Streptomyces* that causes plant scab disease [49]. This effect is thought to be resulted from the role of *AtPam16L2* in plant immunity. However, further study is needed to determine the functions of the Pams by additional biological experiments. All the *Pam* gene function study we carried out is based on *Arabidopsis*, further in-depth analysis in other plants is extremely essential in the future plan.

## Conclusions

The mechanism of Pam18 and Pam16 functioning in protein translocation of mitochondria in yeast has been well-studied recently. Both of Pam18 and Pam16 are highly conserved among eukaryotes including plant species; however, their functions in plants remain largely unknown. In this study, thirty-one Pam18 and twenty-six Pam16 proteins from twelve plant species were identified and further analyzed for their properties using bioinformatics strategies. The highly conserved Pam18 and Pam16 orthologues were reconfirmed by multiple sequence alignment. Results depicted that possibly Pam18 and Pam16 can also form a heterodimer through their J-type domains since the key amino acid residues and motifs are identical except OsPam18L5. Based on hydrophobicity analysis, J-type domains are found in the hydrophilic region, so the heterodimer may be formed in a hydrophilic environment. N-myristoylation sites of Pam18s and casein kinase II phosphorylation sites of Pam16s are more abundant, which might tend to be important functional sites. Some Pam18s and Pam16s contain transmembrane regions at their N-terminal region. It is most likely that some Pam18s and Pam16s localize to the mitochondria since not all but many Pam18s and Pam16s were predicted so according to three predictors. Taken together, it is possible that some Pam18 and Pam16 orthologues in plants could also form heterodimers and regulate protein translocation in mitochondria. In the model plant *Arabidopsis*, *AtPam* genes were expressed higher at senescence stage, suggesting *AtPam* genes probably regulate plant senescence. Real-time qRT-PCR results depicted that salt stress can induce the expression of *AtPam18L3*, *AtPam16L1* and *AtPam16L2*. The expression of *AtPam18L1* and *AtPam16L1* were up-regulated by drought stress. Gene *AtPam18L1*, *AtPam18L2*, *AtPam18L3*, *AtPam16L1* and *AtPam16L2* may play a role in heat stress responses. It is therefore thoughtfully concluded that these findings provide a better understanding of Pam18 and Pam16 in plants.

## Supporting Information

Table S1 **Putative orthologues of ScPam18 and ScPam16 in twelve plant species.**
(DOC)

Figure S1 **Schematic representation and transmembrane region of ScPam18 and ScPam16.**
(DOC)

## References

[1] Mcbride MH, Neuspiel M, Wasiak S (2006) Mitochondria: more than just a powerhouse. Curr Biol 16: 551-560. doi:10.1016/j.sbi.2006.06.011. PubMed: 16860735. 16860735

[2] Ernster L, Schatz G (1981) Mitochondria: a historical review. J Cell Biol 91: 227-255. doi:10.1083/jcb.91.3.227s. PubMed: 7298718. 7033239 PMC2112799

[3] Newmeyer DD, Ferguson-Miller S (2003) Mitochondria: releasing power for life and unleashing the machineries of death. Cell 112: 481-490. doi:10.1016/S0092-8674(03)00116-8. PubMed: 12600312. 12600312

[4] Jensen RE, Johnson AE (2001) Opening the door to mitochondrial protein import. Nat Struct Biol 8: 1008-1010. doi:10.1038/nsb1201-1008. PubMed: 11723465. 11723465

[5] Endo T, Kohda D (2002) Functions of outer membrane receptors in mitochondrial protein import. Biochim Biophys Acta 1592: 3-14. doi:10.1016/S0167-4889(02)00259-8. PubMed: 12191763. 12191763

[6] Neupert W, Brunner M (2002) The protein import motor of mitochondria. Nat Rev Mol Cell Biol 3: 555-565. doi:10.1038/nrm878. PubMed: 12154367. 12154367

[7] Truscott KN, Brandner K, Pfanner N (2003) Mechanisms of protein import into mitochondria. Curr Biol 13: 326-337. doi:10.1016/S0960-9822(03)00239-2. PubMed: 12699647. 12699647

[8] Frazier AE, Dudek J, Guiard B, Voos W, Li Y et al. (2004) Pam16 has an essential role in the mitochondrial protein import motor. Nat Struct Mol Biol 11: 226-233. doi:10.1038/nsmb735. PubMed: 14981507. 14981507

[9] van der Laan M, Hutu DP, Rehling P (2010) On the mechanism of preprotein import by the mitochondrial presequence translocase. Biochim Biophys Acta 1803: 732-739. doi:10.1016/j.bbamcr.2010.01.013. PubMed: 20100523. 20100523

[10] Marom M, Azem A, Mokranjac D (2011) Understanding the molecular mechanism of protein translocation across the mitochondrial inner membrane: still a long way to go. Biochim Biophys Acta 1808: 990-1001. doi:10.1016/j.bbamem.2010.07.011. PubMed: 20646995. 20646995

[11] Glick BS (1995) Can Hsp70 proteins act as force-generating motors? Cell 80: 11-14. doi:10.1016/0092-8674(95)90444-1. PubMed: 7813006. 7813006

[12] Pilon M, Schekman R (1999) Protein translocation: how Hsp70 pulls it off. Cell 97: 679-682. doi:10.1016/S0092-8674(00)80780-1. PubMed: 10380919. 10380919

[13] Matouschek A, Pfanner N, Voos W (2000) Protein unfolding by mitochondria: the Hsp70 import motor. EMBO Rep 1: 404-410. doi:10.1093/embo-reports/kvd093. PubMed: 11258479. 11258479 PMC1083766

[14] Mokranjac D, Neupert W (2010) The many faces of the mitochondrial TIM23 complex. Biochim Biophys Acta 1797: 1045-1054. doi:10.1016/j.bbabio.2010.01.026. PubMed: 20116361. 20116361

[15] Endo T, Yamano K, Kawano S (2011) Structural insight into the mitochondrial protein import system. Biochim Biophys Acta 1808: 955-970. doi:10.1016/j.bbamem.2010.07.018. PubMed: 20655871. 20655871

[16] Schilke BA, Hayashi M, Craig EA (2012) Genetic analysis of complex interactions among components of the mitochondrial import motor and translocon in *Saccharomyces* *cerevisiae* . Genetics 190: 1341-1353. doi:10.1534/genetics.112.138743. PubMed: 22298705. 22298705 PMC3316647

[17] van der Laan M, Chacinska A, Lind M, Perschil I, Sickmann A et al. (2005) Pam17 is required for architecture and translocation activity of the mitochondrial protein import motor. Mol Cell Biol 25: 7449-7458. doi:10.1128/MCB.25.17.7449-7458.2005. PubMed: 16107694. 16107694 PMC1190294

[18] D'Silva PR, Schilke B, Walter W, Craig EA (2005) Role of Pam16's degenerate J domain in protein import across the mitochondrial inner membrane. Proc Natl Acad Sci USA 102: 12419-12424. 16105940 10.1073/pnas.0505969102 PMC1194952

[19] Pais JE, Schilke B, Craig EA (2011) Reevaluation of the role of the Pam18:Pam16 interaction in translocation of proteins by the mitochondrial Hsp70-based import motor. Mol Cell Biol 22: 4740-4749. doi:10.1091/mbc.E11-08-0715. PubMed: 22031295. PMC3237618 22031295

[20] Mokranjac D, Bourenkov G, Hell K, Neupert W, Groll M (2006) Structure and function of Tim14 and Tim16, the J and J-like components of the mitochondrial protein import motor. EMBO J 25: 4675-4685. 16977310 10.1038/sj.emboj.7601334 PMC1590002

[21] Koehler CM (2004) The small Tim proteins and the twin Cx3C motif. Trends Biochem Sci 29: 1-4. doi:10.1016/j.tibs.2003.11.003. PubMed: 14729324. 14729324

[22] Rehling P, Brandner K, Pfanner N (2004) Mitochondrial import and the twin-pore translocase. Nat Rev Mol Cell Biol 5: 519-530. doi:10.1038/nrm1426. PubMed: 15232570. 15232570

[23] Elsner S, Simian D, Iosefson O, Marom M, Azem A (2009) The mitochondrial protein translocation motor: structural conservation between the human and yeast Tim14/Pam18-Tim16/Pam16 co-chaperones. Int J Mol Sci 10: 2041-2053. doi:10.3390/ijms10052041. PubMed: 19564938. 19564938 PMC2695266

[24] Paul P, Simm S, Blaumeiser A, Scharf KD, Fragkostefanakis S et al. (2013) The protein translocation systems in plants- composition and variability on the example of *Solanum* *lycopersicum* . BMC Genomics 14: 189-205. doi:10.1186/1471-2164-14-189. PubMed: 23506162. 23506162 PMC3610429

[25] Altschul SF, Gish W, Miller W, Myers EW, Lipman DJ (1990) Basic local alignment search tool. J Mol Biol 215: 403-410. doi:10.1016/S0022-2836(05)80360-2. PubMed: 2231712. 2231712

[26] Andolfo G, Sanseverino W, Rombauts S, van de Peer Y, Bradeen JM et al. (2012) Overview of tomato (*Solanum* *lycopersicum*) candidate pathogen recognition genes reveals important Solanum R locus dynamics. New Phytol 197: 223-237. PubMed: 23163550. 23163550 10.1111/j.1469-8137.2012.04380.x

[27] Tamura K, Peterson D, Peterson N, Stecher G, Nei M et al. (2011) MEGA5: molecular evolutionary genetics analysis using maximum likelihood, evolutionary distance, and maximum parsimony methods. Mol Biol Evol 28: 2731-2739. doi:10.1093/molbev/msr121. PubMed: 21546353. 21546353 PMC3203626

[28] Kyte J, Doolittle RF (1982) A simple method for displaying the hydrophobic character of a protein. J Mol Biol 157: 105-142. doi:10.1016/0022-2836(82)90515-0. PubMed: 7108955. 7108955

[29] Yuan Z, Mattick JS, Teasdale RD (2004) SVMtm: support vector machines to predict transmembrane segments. J Comput Chem 25: 632-636. doi:10.1002/jcc.10411. PubMed: 14978706. 14978706

[30] de Castro E, Sigrist CJ, Gattiker A, Bulliard V, Langendijk-Genevaux PS et al. (2006) ScanProsite: detection of PROSITE signature matches and ProRule-associated functional and structural residues in proteins. Nucleic Acids Res 34: W362-W365. doi:10.1093/nar/gkl124. PubMed: 16845026. 16845026 PMC1538847

[31] Emanuelsson O, Nielsen H, Brunak S, von Heijne G (2000) Predicting subcellular localization of proteins based on their N-terminal amino acid sequence. J Mol Biol 300: 1005-1016. doi:10.1006/jmbi.2000.3903. PubMed: 10891285. 10891285

[32] Hruz T, Laule O, Szabo G, Wessendorp F, Bleuler S et al. (2008) Genevestigator v3: a reference expression database for the meta-analysis of transcriptomes. Adv Bioinformatics, 2008: 420747. doi:10.1155/2008/420747. PubMed: 19956698. 19956698 PMC2777001

[33] Hamilton JP, Buell CR (2012) Advances in plant genome sequencing. Plant J 70: 177-190. doi:10.1111/j.1365-313X.2012.04894.x. PubMed: 22449051. 22449051

[34] the Tomato Genome Consortium (2012) The tomato genome sequence provides insights into fleshy fruit evolution. Nature 485: 635-641. doi:10.1038/nature11119. PubMed: 22660326. 22660326 PMC3378239

[35] Bies C, Blum R, Dudek J, Nastainczyk W, Oberhauser S et al. (2004) Characterization of pancreatic ERj3p, a homolog of yeast DnaJ-like protein Scj1p. Biol Chem 385: 389-395. PubMed: 15195998. 15195998 10.1515/BC.2004.043

[36] Chacinska A, Lind M, Frazier AE, Dudek J, Meisinger C et al. (2005) Mitochondrial presequence translocase: switching between TOM tethering and motor recruitment involves Tim21 and Tim17. Cell 120: 817-829. doi:10.1016/j.cell.2005.01.011. PubMed: 15797382. 15797382

[37] Bukau B, Weissman J, Horwich A (2006) Molecular chaperones and protein quality control. Cell 125: 443-451. doi:10.1016/j.cell.2006.04.014. PubMed: 16678092. 16678092

[38] Craig EA, Huang P, Aron R, Andrew A (2006) The diverse roles of J-proteins, the obligate Hsp70 co-chaperone. Rev Physiol Biochem Pharmacol 156: 1-21. PubMed: 16634144. 16634144 10.1007/s10254-005-0001-0

[39] Rayon C, Lerouge P, Faye L (1998) The protein N-glycosylation in plants. J Exp Bot 49: 1463-1472. doi:10.1093/jxb/49.326.1463.

[40] Podell S, Gribskov M (2004) Predicting N-terminal myristoylation sites in plant proteins. BMC Genomics 5: 37-52. doi:10.1186/1471-2164-5-37. PubMed: 15202951. 15202951 PMC449705

[41] Huber SC (2007) Exploring the role of protein phosphorylation in plants: from signalling to metabolism. Biochem Soc Trans 35: 28-32. doi:10.1042/BST0350028. PubMed: 17212583. 17212583

[42] Guiboileau A, Sormani R, Meyer C, Masclaux-Daubresse C (2010) Senescence and death of plant organs: nutrient recycling and developmental regulation. C R Biol 333: 382-391. doi:10.1016/j.crvi.2010.01.016. PubMed: 20371113. 20371113

[43] Quirino BF, Noh YS, Himelblau E, Amasino RM (2000) Molecular aspects of leaf senescence. Trends Plant Sci 5: 278-282. doi:10.1016/S1360-1385(00)01655-1. PubMed: 10871899. 10871899

[44] Yoshida S (2003) Molecular regulation of leaf senescence. Curr Opin Plant Biol 6: 79-84. doi:10.1016/S1369526602000092. PubMed: 12495755. 12495755

[45] Yamaguchi-Shinozaki K, Shinozaki K (2006) Transcriptional regulatory networks in cellular responses and tolerance to dehydration and cold stresses. Annu Rev Plant Biol 57: 781-803. doi:10.1146/annurev.arplant.57.032905.105444. PubMed: 16669782. 16669782

[46] Maxwell DP, Nickels R, McIntosh L (2002) Evidence of mitochondrial involvement in the transduction of signals required for the induction of genes associated with pathogen attack and senescence. Plant J 29: 269-279. doi:10.1046/j.1365-313X.2002.01216.x. PubMed: 11844105. 11844105

[47] Dutilleul C, Garmier M, Noctor G, Mathieu C, Chétrit P et al. (2003) Leaf mitochondria modulate whole cell redox homeostasis, set antioxidant capacity, and determine stress resistance through altered signaling and diurnal regulation. Plant Cell 15: 1212-1226. doi:10.1105/tpc.009464. PubMed: 12724545. 12724545 PMC153727

[48] Ho LH, Giraud E, Uggalla V, Lister R, Clifton R et al. (2008) Identification of regulatory pathways controlling gene expression of stress-responsive mitochondrial proteins in *Arabidopsis* . Plant Physiol 147: 1858-1873. doi:10.1104/pp.108.121384. PubMed: 18567827. 18567827 PMC2492625

[49] Scheible WR, Fry B, Kochevenko A, Schindelasch D, Zimmerli L et al. (2003) An Arabidopsis mutant resistant to thaxtomin A, a cellulose synthesis inhibitor from *Streptomyces* species. Plant Cell 15: 1781-1794. doi:10.1105/tpc.013342. PubMed: 12897252. 12897252 PMC167169

