# Supplementary material for: Comparative Analysis of Putative Orthologues of Mitochondrial Import Motor Subunit: Pam18 and Pam16 in Plants
Source: PLoS One. 2013 Oct 23;8(10):e78400. doi: 10.1371/journal.pone.0078400 (PMC3806816; doi:10.1371/journal.pone.0078400)
Supplement: Table S1 — Putative orthologues of ScPam18 and ScPam16 in twelve plant species. (DOC) [file pone.0078400.s001.doc]

**Table S1.** Putative orthologues of ScPam18 and ScPam16 in twelve plant species.

| **Plant species** | **Protein**  **name** | **Protein**  **length (aa)** | **Maximum**  **identity (%)** | **E**  **value** | **Accession number** |
| --- | --- | --- | --- | --- | --- |
| *Arabidopsis thaliana* (At) | AtPam18L1 | 112 | 57 | 1e-18 | NP_565824.1 |
|  | AtPam18L2 | 112 | 54 | 2e-17 | NP_566352.1 |
|  | AtPam18L3 | 112 | 52 | 8e-17 | NP_195923.1 |
|  | AtPam16L1 | 113 | 38 | 9e-13 | NP_568943.1 |
|  | AtPam16L2 | 116 | 35 | 5e-12 | NP_567078.1 |
| *Oryza sativa* (Os) | OsPam18L1 | 76 | 57 | 2e-17 | EEE53903.1 |
|  | OsPam18L2 | 147 | 57 | 3e-17 | EEC81671.1 |
|  | OsPam18L3 | 114 | 57 | 3e-17 | NP_001059099.1 |
|  | OsPam18L4 | 111 | 49 | 1e-15 | NP_001051437.1 |
|  | OsPam18L5 | 103 | 42 | 2e-10 | EAY92033.1 |
|  | OsPam16L1 | 113 | 37 | 3e-11 | NP_001172755.1 |
|  | OsPam16L2 | 116 | 35 | 5e-10 | NP_001064869.1 |
|  | OsPam16L3 | 345 | 36 | 2e-08 | EEC67178.1 |
| *Zea mays* (Zm) | ZmPam18L1 | 112 | 57 | 1e-18 | NP_001146932.1 |
|  | ZmPam18L2 | 125 | 57 | 3e-18 | ACG30878.1 |
|  | ZmPam18L3 | 113 | 54 | 2e-17 | NP_001148450.1 |
|  | ZmPam16L1 | 113 | 38 | 4e-12 | ACG36316.1 |
|  | ZmPam16L2 | 116 | 36 | 6e-11 | NP_001152588.1 |
| *Glycine max* (Gm) | GmPam18L1 | 110 | 55 | 1e-17 | NP_001237577.1 |
|  | GmPam18L2 | 112 | 55 | 1e-17 | XP_003524634.1 |
|  | GmPam16L1 | 116 | 36 | 8e-14 | XP_003527068.1 |
|  | GmPam16L2 | 116 | 34 | 4e-11 | NP_001235734.1 |
| [*Sorghum bicolor*](http://www.ncbi.nlm.nih.gov/entrez/query.fcgi?db=genomeprj&cmd=Retrieve&dopt=Overview&list_uids=10785) (Sb) | SbPam18L1 | 112 | 57 | 7e-19 | XP_002455169.1 |
|  | SbPam18L2 | 132 | 54 | 2e-17 | XP_002466363.1 |
|  | SbPam16L1 | 113 | 38 | 4e-12 | XP_002459141.1 |
|  | SbPam16L2 | 116 | 36 | 3e-11 | XP_002467071.1 |
| *Solanum lycopersicum* (Sl) | SlPam18L1 | 112 | 62 | 2e-20 | XP_004246685.1 |
|  | SlPam18L2 | 112 | 57 | 3e-19 | XP_004249683.1 |
|  | SlPam18L3 | 109 | 56 | 4e-18 | XP_004241472.1 |
|  | SlPam16L1 | 114 | 34 | 2e-12 | XP_004244470.1 |
|  | SlPam16L2 | 114 | 35 | 7e-12 | XP_004241664.1 |
|  | SlPam16L3 | 146 | 30 | 2e-08 | XP_004253402.1 |
|  | SlPam16L4 | 288 | 33 | 4e-06 | XP_004245437.1 |
| [*Medicago truncatula*](http://www.ncbi.nlm.nih.gov/entrez/query.fcgi?db=genomeprj&cmd=Retrieve&dopt=Overview&list_uids=9508) (Mt) | MtPam18L1 | 110 | 56 | 5e-18 | AFK46576.1 |
|  | MtPam16L1 | 115 | 34 | 4e-12 | AFK49063.1 |
| *Populus trichocarpa* (Pt) | PtPam18L1 | 112 | 57 | 2e-18 | XP_002311111.1 |
|  | PtPam18L2 | 112 | 45 | 4e-18 | XP_002326335.1 |
|  | PtPam18L3 | 112 | 52 | 8e-18 | XP_002318329.1 |
|  | PtPam16L1 | 116 | 35 | 3e-13 | XP_002321119.1 |
|  | PtPam16L2 | 114 | 37 | 3e-12 | XP_002318780.1 |
| [*Vitis vinifera*](http://www.ncbi.nlm.nih.gov/entrez/query.fcgi?db=genomeprj&cmd=Retrieve&dopt=Overview&list_uids=12992) (Vv) | VvPam18L1 | 115 | 57 | 3e-18 | CAN77055.1 |
|  | VvPam18L2 | 110 | 57 | 5e-18 | XP_002279532.1 |
|  | VvPam18L3 | 112 | 52 | 1e-17 | XP_002269777.1 |
|  | VvPam18L4 | 166 | 52 | 8e-17 | CBI35310.3 |
|  | VvPam16L1 | 120 | 32 | 2e-10 | XP_002267730.1 |
|  | VvPam16L2 | 253 | 34 | 3e-09 | CBI39152.3 |
|  | VvPam16L3 | 108 | 34 | 5e-09 | CAN73075.1 |
| *Picea sitchensis* (Ps) | PsPam18L1 | 112 | 56 | 3e-18 | ABK25685.1 |
|  | PsPam16L1 | 115 | 35 | 5e-13 | ABK21068.1 |
| [*Brachypodium distachyon*](http://www.ncbi.nlm.nih.gov/entrez/query.fcgi?db=genomeprj&cmd=Retrieve&dopt=Overview&list_uids=18703) (Bd) | BdPam18L1 | 112 | 57 | 5e-18 | XP_003557436.1 |
|  | BdPam18L2 | 111 | 51 | 2e-17 | XP_003558556.1 |
|  | BdPam18L3 | 112 | 54 | 3e-16 | XP_003567664.1 |
|  | BdPam16L1 | 113 | 39 | 4e-13 | XP_003565121.1 |
|  | BdPam16L2 | 116 | 36 | 6e-11 | XP_003574068.1 |
| *Physcomitrella patens* (Pp) | PpPam18L1 | 112 | 55 | 1e-18 | XP_001772964.1 |
|  | PpPam16L1 | 115 | 34 | 3e-10 | XP_001761102.1 |
|  | PpPam16L2 | 117 | 31 | 5e-10 | XP_001757252.1 |
