# Supplementary material for: Comparative Analysis of Putative Orthologues of Mitochondrial Import Motor Subunit: Pam18 and Pam16 in Plants
Source: PLoS One. 2013 Oct 23;8(10):e78400. doi: 10.1371/journal.pone.0078400 (PMC3806816; doi:10.1371/journal.pone.0078400)
Supplement: Figure S1 — Schematic representation and transmembrane region of ScPam18 and ScPam16. (DOC) [file pone.0078400.s002.doc]

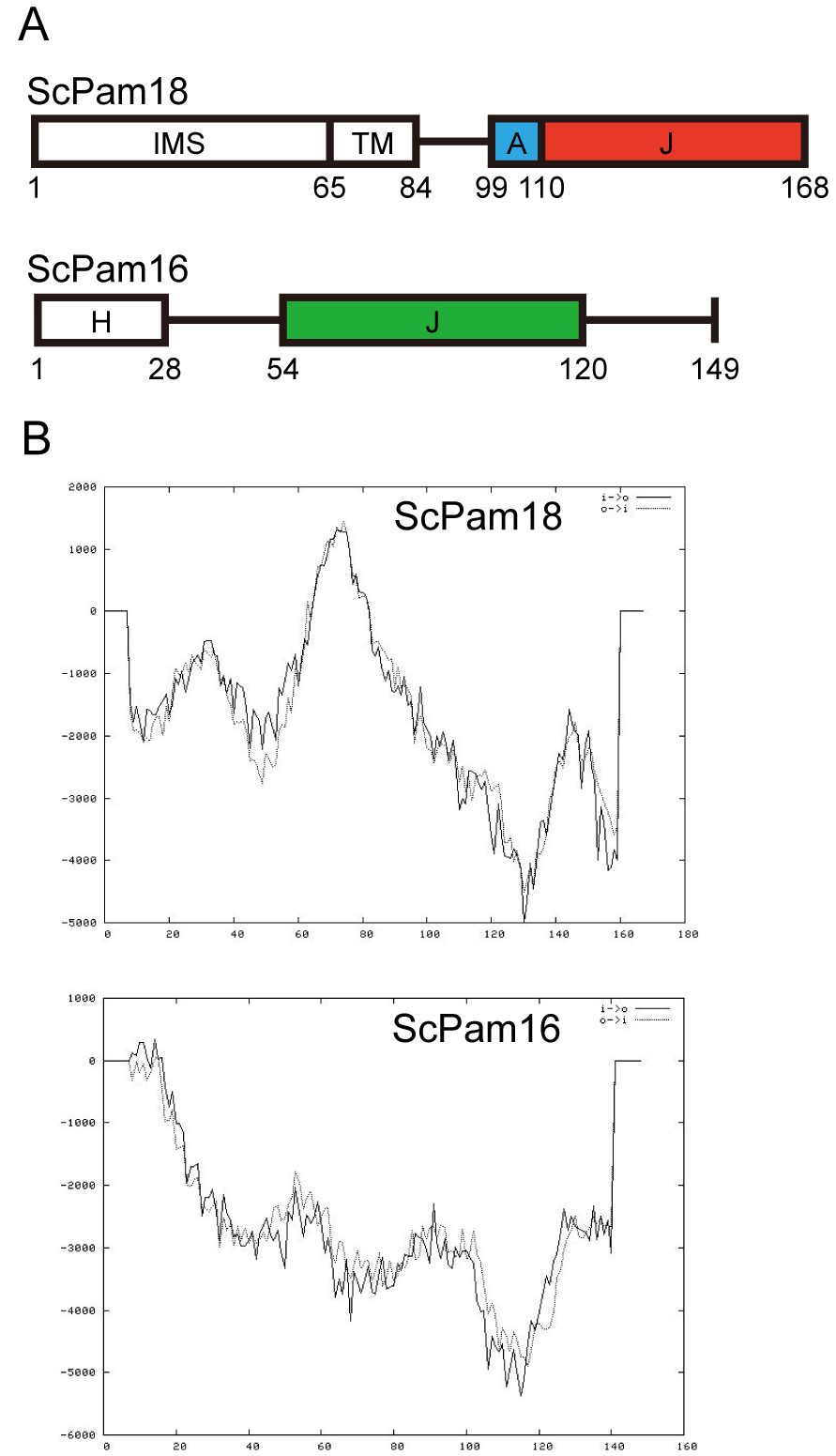


**Figure S1. Schematic representation and transmembrane region of ScPam18 and ScPam16.**

A. ScPam18: amino acids (aa) 1–64, intermembrane space (IMS); aa 65–83, transmembrane (TM); aa 99–109, arm (A); aa 110–168, J-domain (J). ScPam16: aa 1–27, hydrophobic (H); aa 54–119, J-like domain (J). B. Prediction of transmembrane regions in ScPam18, ScPam16.
